# PremPDI estimates and interprets the effects of missense mutations on protein-DNA interactions

**DOI:** 10.1371/journal.pcbi.1006615

**Published:** 2018-12-11

**Authors:** Ning Zhang, Yuting Chen, Feiyang Zhao, Qing Yang, Franco L. Simonetti, Minghui Li

**Affiliations:** 1 School of Biology and Basic Medical Sciences, Soochow University, Suzhou, China; 2 Institute Leloir Foundation, Buenos Aires, Argentina; Clemson University, UNITED STATES

## Abstract

Protein-DNA interactions play important roles in regulations of many vital cellular processes, including transcription, translation, DNA replication and recombination. Sequence variants occurring in these DNA binding proteins that alter protein-DNA interactions may cause significant perturbations or complete abolishment of function, potentially leading to diseases. Developing a mechanistic understanding of impacts of variants on protein-DNA interactions becomes a persistent need. To address this need we introduce a new computational method PremPDI that predicts the effect of single missense mutation in the protein on the protein-DNA interaction and calculates the quantitative binding affinity change. The PremPDI method is based on molecular mechanics force fields and fast side-chain optimization algorithms with parameters optimized on experimental sets of 219 mutations from 49 protein-DNA complexes. PremPDI yields a very good agreement between predicted and experimental values with Pearson correlation coefficient of 0.71 and root-mean-square error of 0.86 kcal mol^-1^. The PremPDI server could map mutations on a structural protein-DNA complex, calculate the associated changes in binding affinity, determine the deleterious effect of a mutation, and produce a mutant structural model for download. PremPDI can be applied to many tasks, such as determination of potential damaging mutations in cancer and other diseases. PremPDI is available at http://lilab.jysw.suda.edu.cn/research/PremPDI/.

## Author summary

Developing methods for accurate prediction of effects of amino acid substitutions on protein-DNA interactions is important for a wide range of biomedical applications such as understanding disease-causing mechanism of missense mutations and guiding protein engineering. Very few methods have been developed for predicting the effects of mutations on protein-DNA binding affinity. Here we report a new computational method, PRedicts the Effects of single Mutations on Protein-DNA Interactions (PremPDI). The core of the PremPDI method is based on molecular mechanics force fields and fast side-chain optimization algorithms that makes the PremPDI algorithm efficient and being fast enough to handle large number of cases. The performance of the PremPDI protocol was tested against experimentally determined binding free energy changes of 219 mutations from 49 protein-DNA complexes and yields very good correlation coefficient. The PremPDI webserver is available to the community at http://lilab.jysw.suda.edu.cn/research/PremPDI/.

## Introduction

There has been a rapid development of genome-wide techniques in the last decade along with significant lowering of the cost of gene sequencing, which generated widely available genomic data. However, the interpretation of genomic data and prediction of the association of genetic variations with diseases and phenotypes still require significant improvement [1]. Crucial prerequisite for proper biological function is a protein’s ability to establish highly selective interactions with macromolecular partners. Protein-DNA interactions play important roles in regulations of many vital cellular processes, including transcription, translation, DNA replication, repair and recombination. Sequence variants occurring in these DNA binding proteins that alter protein-DNA interactions may cause significant perturbations or complete abolishment of function, potentially leading to many diseases, such as cancer and heart diseases [2–4]. One possible way to assess the effect of a mutation on protein-DNA interaction is to experimentally measure the binding affinity change. However, while site-directed mutagenesis methods are inexpensive and fast, surface plasmon resonance [5], isothermal titration calorimetry [6], FRET [7] and other methods used to measure binding affinity can be time-consuming and costly. Therefore, the development of reliable computational approaches to predict the effects of missense mutations on proteins and their complexes would give us important clues for identifying functionally important missense mutations, understanding the molecular mechanisms of diseases and facilitating their treatment and prevention.

With recent rapid advances in computational biology, many approaches have been developed to offer a phenotypic classification of mutations into damaging and neutral categories [8–10], to calculate the impact of mutations on protein stability [11–13] and protein-protein interactions [14–18]. Previously, we developed two methods for predicting the effect of single mutation on protein-protein binding affinity change. One used modified MM/PBSA, statistical scoring energy functions and structure minimization protocol with explicit solvent model [17]. The other updated method of MutaBind [14], which combined additional features and used a 100-step energy minimization in the gas phase that considerably increases the prediction accuracy and calculation speed. Our method was applied to predict the effects of cancer mutations on the binding between CBL ubiquitin ligase and E2 conjugating enzyme, where predicted binding affinity changes were successfully compared with the experiments using cancer and non-cancer cell lines [19]. However, very few methods can predict the effects of mutations on protein-DNA binding affinity [20]. Very recently, two prediction methods with servers, mCSM-NA [21] and SAMPDI [22], were proposed for performing this task. mCSM-NA relies on graph-based signatures and can predict the effect of single mutation on protein-DNA and protein-RNA binding, while SAMPDI combines modified MM/PBSA based energy terms with additional knowledge-based terms for predicting the protein-DNA binding affinity change upon single mutation. As we know, machine learning methods that use different features and training sets may produce different performances on diverse mutations and complexes[23]. Therefore, more fast and accurate computational methods need to be developed for increasing the range of applications on different kinds of complexes and mutations and explaining the mechanisms, such as the molecular mechanisms of disease progression caused by mutations.

To address this need we present a new computational method and webserver, PremPDI (http://lilab.jysw.suda.edu.cn/research/PremPDI/) which is based on molecular mechanics force fields and fast side-chain optimization algorithms. PremPDI can evaluate the effects of sequence variants and disease mutations (both interfacial and non-interfacial mutations) on protein-DNA interactions; calculate the quantitative change in binding affinity upon single mutation; assess deleterious effects and produce models of mutant complexes. PremPDI is validated using different types of cross-validation and is compared with two other methods using a variety of training and test sets. PremPDI can be applied to many tasks, including finding potential driver missense mutations in cancer, investigating the effects of sequence variations on protein fitness in evolution and protein design.

## Methods

### Compilation of experimental datasets of mutations

ProNIT database [24] includes experimentally measured values of changes in binding free energies upon single and multiple amino acid substitutions (called “mutations” hereafter) derived from the scientific literatures for protein-nucleic acid complexes with experimentally determined structures. dbAMEPNI database [25], being developed recently, focuses on the effects of single alanine-scanning mutations on the experimentally measured binding affinities between protein and nucleic acid. It comprises a total of 577 mutations with quantitatively characterized thermodynamic effects, among of them 345 were taken from ProNIT database. Both databases were used for compiling the dataset for parameterization of PremPDI. The following criteria were applied in constructing our dataset: removal complexes without wild-type protein structures or with modified residues or nucleotides at the binding interface of protein-DNA; removal mutations for their mutated sites with missing coordinates in the corresponding wild-type complex structures; eliminating ProNIT entries with multiple mutations restricting our set to single mutations. Furthermore, to avoid the inconsistency between nucleic acids used for measuring binding affinity and those for developing prediction model based on complex 3D structures, we carried out the comparison of sequence similarity between the nucleic acids of binding sites observed in the protein-DNA structures and the sequences used in the corresponding experiments. Then the entries with high sequence similarity (80%) for the nucleic acids in the binding interface were kept. ProNIT database includes the sequences of DNA used for measuring binding affinity, while dbAMEPNI database does not. So, we manually compiled them from the corresponding references. There are some entries where several experimental values are available for the same mutation. For these cases that are not drastically different from each other, we used an average value of experimental changes in binding free energy. In addition, 20 mutations from five protein-DNA complexes abstracted from SAMPDI training set [22] were also included in our dataset. As a result, the experimental set used in this study includes 219 single mutations from 49 wild-type protein-DNA complexes (it will be referred to as “Prempdi”) (S1 Table). Only 105 mutations obtained from ProNIT database have the information of experimental pH. Thus, we chose the experimental pH to be neutral assuming that at neutral pH the ionizable residues have default charged states. The number of mutations for each protein-DNA complex is shown in S1 Fig We also compared our dataset with the training datasets used for developing SAMPDI and mCSM methods, and the details are shown in S1 Table.

### Structure optimization protocol

Crystal or NMR structures of wild-type protein-DNA complexes were obtained from the Protein Data Bank (PDB) [26], and biological assembly 1 of crystal structure or the first model of NMR was used as the initial structure. First we introduced a single mutation on the wild-type Protein-DNA complex structure using BuildModel module from FoldX [27] software package. Missing heavy side chain atoms and hydrogen atoms were added for the wild type and mutant using VMD program [28] based on the topology file from the CHARMM36 force field [29]. Then a 100-step energy minimization in the gas phase was carried out for both wild type and mutant using harmonic restraints (with the force constant of 5 kcal mol^-1^ Å^-2^) applied on the backbone atoms of all residues. Minimization was done only for protein-DNA complexes, and protein or nucleic acid structures of binding partners were retained assuming the rigid-body binding. The energy minimization was carried out with NAMD program version 2.12 [30] using the CHARMM36 force field [29]. A 12 Å cutoff distance for nonbonded interactions was applied to the systems. Lengths of hydrogen-containing bonds were constrained by the SHAKE algorithm [31]. The current structure optimization protocol was chosen based on its highest accuracy and speed. The performances for other structure optimization protocols that have been tried are shown in S2 Table. The minimized structures of wild-type and mutant complexes were used for the calculation of energy terms.

### Calculation of binding energy terms

Our goal is to design a method to assess the effects of mutations on protein-DNA binding. Mutations can affect binding in different ways [32]. They may change the components of protein-DNA interaction energies, may affect the solvation of a complex, may change the hydrogen-bond network and may directly disrupt binding hotspot sites [33]. Besides, the interactions between protein and the two types of nucleic acids (DNA and RNA) are also different, which was validated by a detailed computational comparison at the atomic contact level [34]. Here, through analysis of different kinds of protein sequence and structural features (S3 Table shows all features considered in our model selection), we found that nine features contributed significantly to the quality of multiple linear regression model (MLR) for the calculation of ΔΔ*G* value (change in binding affinity upon mutation) affecting protein-DNA interactions (Table 1). The features that contribute significantly to the quality of PremPDI model are described below.

**Table 1:** The p-value and importance of each feature in energy function for binding affinity change determined by multiple linear regression (MLR).

Feature	P-value	Importance
${SA}_{com/p2}^{wt}$	5.74e-09	0.47
ΔΔ***G***_*solv*_	6.34e-08	0.41
${\Delta N}_{Hbond}^{p1-p2}$	5.37e-06	0.33
$\Delta E_{elec}^{mut.(p1-p2)}$	1.98e-07	0.28
$N_{Hbond}^{wt.(site-all)}$	2.33e-06	0.27
***L***_*mut*_	9.42e-04	0.26
$\Delta \Delta E_{vdw}^{site-all}$	4.30e-04	0.18
Δ***E***_*fold*_	3.03e-03	0.17
$\Delta_{location}^{mut}$	2.28e-03	0.17

All features have significant contribution to the quality of the model with p-value < 0.01 (*t*-test). Standardized coefficients are used for describing the importance for MLR.

- ΔΔ*G*_*solv*_ is the difference between polar solvation energies of mutant and wild-type protein-DNA complexes ($\Delta \Delta G_{solv}=\Delta G_{solv}^{mut}-\Delta G_{solv}^{wt}$). $\Delta G_{solv}^{wt}$ and $\Delta G_{solv}^{mut}$ are the differences between polar solvation energies of a complex and each interacting partner (Δ*G* = *G*_*com*_ − *G*_*p*1_ − *G*_*p*2_) (p1: partner1, proteins; p2: partner2, DNA) in water for wild-type and mutant complexes respectively. These terms are calculated from solving the Poisson-Boltzmann equation with PBEQ module [35] of CHARMM program [36]. For the PB calculation, dielectric constants, ε = 2, 6, 10, 14, 18 and 20, were tested using the optimized minimization protocol and energy function. As a result, ε = 2 for the protein interior and ε = 80 for the exterior aqueous environment were used for polar solvation energy calculations in our energy model with the best performance (the testing results using different dielectric constants are shown in S4 Table). The ion concentration of zero was used for energy calculation[17].
- $\Delta \Delta E_{vdw}^{site-all}$ is the difference between Van der Waals interaction energies of mutant and wild type ($\Delta \Delta E_{vdw}^{site-all}=\Delta E_{vdw}^{mut.\left(site-all\right)}-\Delta E_{vdw}^{wt.\left(site-all\right)}$). $\Delta E_{vdw}^{wt.(site-all)}$ and $\Delta E_{vdw}^{mut.(site-all)}$ are Van der Waals interaction energies between residue in the mutated site and the rest of protein-DNA complex located within 10 Å from it for wild-type and mutant complexes respectively. They are calculated using ENERGY module of CHARMM program [36].
- $\Delta E_{elec}^{mut.(p1-p2)}$ is electrostatic interaction energy between protein and DNA within 10 Å from each other in mutant. They are calculated using ENERGY module of CHARMM program [36].
- ${\Delta N}_{Hbond}^{p1-p2}\;and\;N_{Hbond}^{wt.(site-all)}:$ ${\Delta N}_{Hbond}^{p1-p2}$ is the difference between the number of hydrogen bonds formed in mutant and wild-type protein-DNA complexes ($\Delta N_{Hbond}^{p1-p2}=N_{Hbond}^{mut.\left(p1-p2\right)}-N_{Hbond}^{wt.\left(p1-p2\right)}$). $N_{Hbond}^{wt.(p1-p2)}$ and $N_{Hbond}^{mut.(p1-p2)}$ terms account for the number of hydrogen bonds formed between protein and DNA for wild-type and mutant complexes respectively; $N_{Hbond}^{wt.(site-all)}$ is the number of hydrogen bonds formed between residue in the mutated site and the rest of wild-type protein-DNA complex. Hydrogen bonds are identified with the CORMAN command of CHARMM program using the following criteria: the maximum distance between acceptor and hydrogen is 2.5 Å and the minimum angle of donor−hydrogen−acceptor is 90°.
- ${SA}_{com/p2}^{wt}$ is the ratio of ${SA}_{com}^{wt}$ and ${SA}_{p2}^{wt}.\;{SA}_{com}^{wt}$ and ${SA}_{p2}^{wt}$ are the solvent accessible surface areas of complex and DNA respectively for wild type. Solvent accessible surface area is calculated using SASA module of CHARMM program.
- $\Delta_{location}^{mut}$ is equal to 1 if the mutation occurs on protein-DNA interface, otherwise it is 0. We define a residue to be located on a protein-DNA interface if residue’s solvent accessibility in the complex is lower than in the corresponding unbound partners.
- Δ*E*_*fold*_ is a pairwise statistical potential for protein folding which was obtained from an optimization procedure that maximizes thermodynamic stability for all proteins simultaneously [37]. It is obtained from Amino Acid Index Database with identifier of MIRL960101 (AAindex, http://www.genome.jp/aaindex/).
- *L*_*mut*_ *is* the length of mutated protein chain.

## Results and discussion

### Model training through multiple linear regression

**Fig 1:**
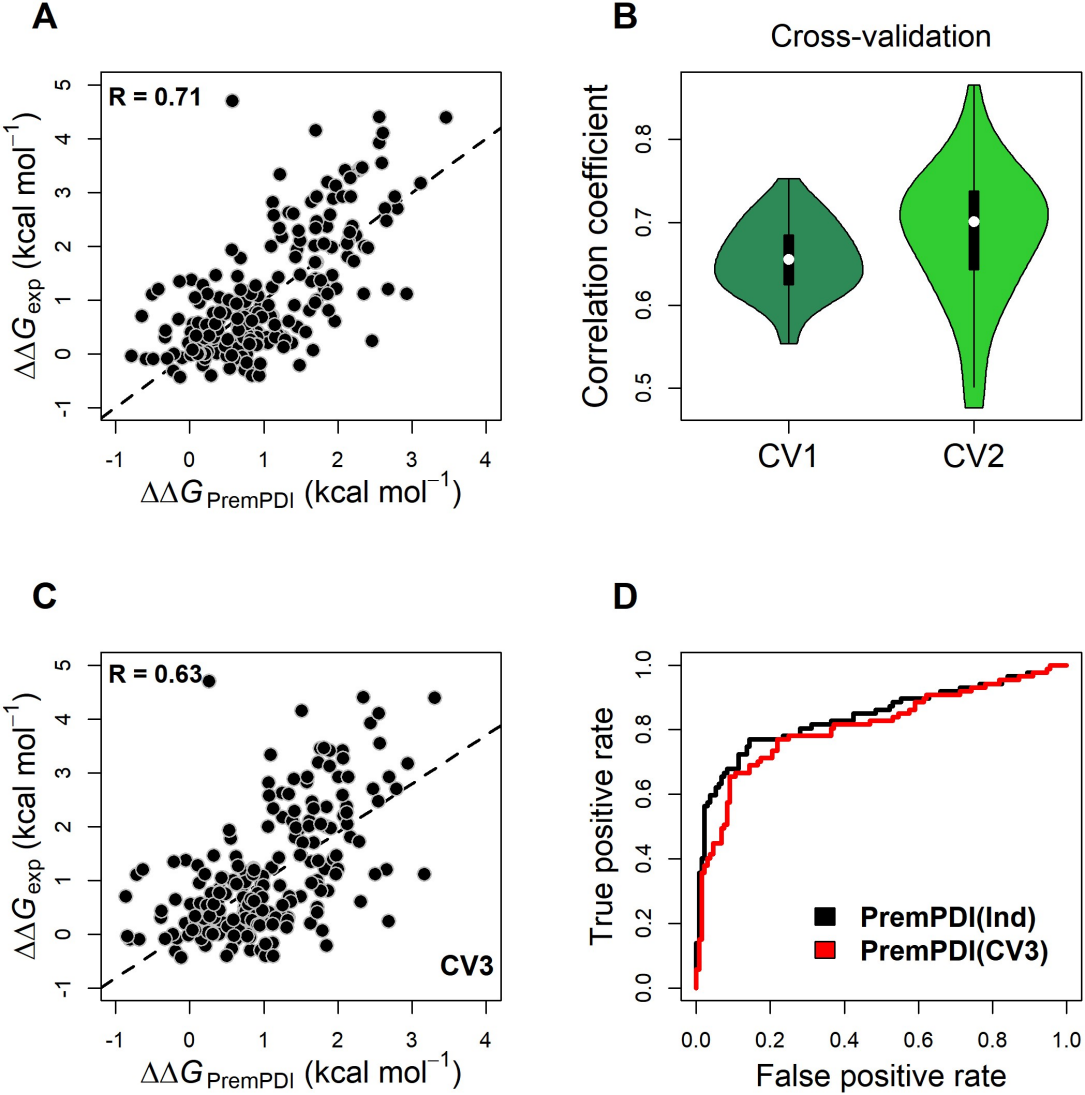
PremPDI performance. Pearson correlation coefficients between experimental and calculated changes in binding free energies (ΔΔ*G*) for “Prempdi” training/test set (a), for two types of cross-validation (CV1 and CV2) (b) and for “leave-one-complex-out” cross-validation (CV3) (c). ROC curves for predictions of deleterious mutations applied on “Prempdi” set (d).

The p-value and contribution of each term to the PremPDI model are shown in Table 1, and all terms contribute significantly to the energy model with p-values less than 0.01. If we train and test our model on the ‘Prempdi’ set, the Pearson correlation coefficient between experimental and calculated changes in binding free energies is R = 0.71 (Fig 1a and Table 2) and the corresponding root-mean-square error (RMSE) is 0.86 (Table 2). Among 219 mutations in “Prempdi” dataset, 179 ones belong to alanine-scanning single mutations defined as substitutions of residues into alanine and 134 ones located on the interfaces of protein-DNA complexes according to our definition (see Method‘ section). The results show that our model does not present bias to alanine-scanning mutations and yields good performance for non-alanine-scanning mutations with R = 0.64 and RMSE = 0.81 (Table 2). As was shown previously [14,17], mutations located on the interface region present average larger effects on protein-protein interactions and are better predicted compared to non-interface mutations. In this study, PremPDI yields statistically significant correlation (p-value < 0.01) in predicting non-interfacial mutations and the correlation reaches value as high as 0.69 and RMSE is 0.85. We also tried several other machine learning methods such as random forest, support vector machine and neural network to build our model using these nine features. Cross-validation and leave one complex validation that will be discussed in the next section show that multiple linear regression represents the best performance.

**Table 2:** PremPDI performance.

Test set	Method	R	RMSE (kcal mol^-1^)	Slope
Prempdi	PremPDI	0.71	0.86	1
	PremPDI (CV1)	0.68	0.90	0.94
	PremPDI (CV2)	0.68	0.90	0.95
	PremPDI (CV3)	0.63	0.95	0.90
Alanine-scanning mutations	PremPDI	0.68	0.87	0.97
	PremPDI (CV3)	0.58	0.96	0.87
Non-Alanine- scanning mutations	PremPDI	0.64	0.81	0.88
	PremPDI (CV3)	0.58	0.88	0.72
Interfacial mutations	PremPDI	0.71	0.86	1.01
	PremPDI (CV3)	0.64	0.95	0.89
Non-interfacial mutations	PremPDI	0.69	0.85	0.98
	PremPDI (CV3)	0.59	0.95	0.91

R: Pearson correlation coefficient between experimental and predicted ΔΔG values. RMSE: root-mean square error. The last column shows the slope of the regression line between experimental and predicted ΔΔG values. All correlation coefficients are statistically significantly different from zero (P-value << 0.01). CV1 and CV2 results for “Alanine-scanning mutations”, “Non-Alanine-scanning mutations”, “Interfacial mutations” and “Non-interfacial mutations” test sets are shown in S5 Table.

In addition, we performed multicollinearity analysis to investigate the linear association across each feature. Pearson correlation matrixes and variance inflation factors (VIF) for the energy features in PremPDI are shown in S6 Table. The results show that ΔΔ*G*_*solv*_ has relatively strong correlation with ${\Delta N}_{Hbond}^{p1-p2}$ (R = -0.71), ${SA}_{com/p2}^{wt}$ has relatively strong correlation with *L*_*mut*_ with R of 0.74, and the rest of the correlations are either small or are not significantly different from zero. The VIFs of all features are less than three representing relatively low multicollinearity. We removed highly correlated features from our energy function that results in decrease of prediction accuracy. For instance, removal ${\Delta N}_{Hbond}^{p1-p2}$ from PremPDI MLR model leads to the decrease of correlation from 0.71 to 0.68. Thus, all nine features were kept in our final model to achieve the optimal performance.

PremPDI takes about five minutes to perform calculations for a single mutation in a protein-DNA complex with 300 residues and 30 nucleotides running on a single processor core, and it requires additional two-to-three minutes for each additional mutation per complex.

### Evaluating the performance of PremPDI using cross-validation and leave one complex validation

Our goal is to construct a computational method that can achieve a high prediction accuracy for large and diverse sets of single mutations. In many cases, overfitting may occur when the parameters of computational methods are tuned to minimize the mean square deviations of predicted from experimental values in the training set, thus leading to the decreased generalized performance [38]. At the same time the training set should be as comprehensive as possible, while in our study the data set used for training and testing is relatively small. To address this issue, we performed three types of cross-validation. In case of “CV1” cross-validation (Fig 1b), 50% mutations selected randomly from “Prempdi” set were used for training and the remaining mutations for testing, the procedure was repeated 50 times. In “CV2” cross-validation we randomly chose 80% of all mutations as training and used the remaining 20% mutations for testing, also repeated 50 times. The average Pearson correlation coefficient is R = 0.68 for both “CV1” and “CV2” with small standard error of 0.06 (Fig 1b). The RMSE is 0.9 kcal mol^-1^ for both cross validations (Table 2).

Since the prediction accuracy of mutational effects largely depends on sequence and structure of a complex, we performed a “leave-one-complex-out” procedure (“CV3” cross-validation). Namely, we trained the parameters on experimental ΔΔ*G* values of mutations from 48 protein-DNA complexes and then applied the model to mutations from the remaining one complex. This procedure was repeated for each complex. The Pearson correlation coefficient between experimental and computed ΔΔ*G* values using this procedure is R = 0.63 with RMSE of 0.95 kcal mol^-1^ (Fig 1c and Table 2). In addition, for alanine-scanning, non-alanine-scanning, interfacial and non-interfacial mutations, they also present relatively high correlation coefficients and low RMSEs in “CV3” cross-validation, especially for interfacial mutations (Table 2).

We also analyzed the variation of the weighting coefficient for each feature in “CV1”, “CV2” and “CV3” cross-validation respectively. The results are shown in S7 Table. The standard deviations of the weighting coefficients are relatively small even for “CV1” cross-validation, 50% mutations from “Prempdi” set were used for training and the remaining mutations for testing, which indicates the variation is not significant across each fold. In addition, the average weighting coefficients in each cross-validation were compared with the weighting coefficients of the final PremPDI model and the results show that the differences for all energy features are very small. All the validations indicate that our PremPDI model does not overfit on its training set and all features have significant contribution to the energy function.

### Evaluating the performance of PremPDI to predict deleterious effects of mutations

Predicting the quantitative values of binding affinity changes is quite challenging. A much easier task, attempted by many studies, is to classify mutations based on their effects into deleterious or neutral. Several thresholds of experimentally determined ΔΔ*G*, 1, 1.5, 2.0 and 2.5 kcal mol^-1^, were tested for defining mutations with deleterious (highly destabilizing) effects (see S2 Fig). The number of mutations in each category is shown in S2a Fig Threshold of 1 kcal mol^-1^ has the most balanced dataset. To quantify the performance of PremPDI scores, we performed Receiver Operating Characteristics (ROC) and precision-recall analyses. Sensitivity or true positive rate was defined as TPR = TP/(TP + FN) and specificity or true negative rate was defined as TNR = 1-FPR = TN/(FP+TN). Additionally, in order to account for imbalances in the labeled dataset, the quality of the predictions was described by Matthews correlation coefficient (MCC), a performance measure which is known to be more robust on unbalanced datasets:

$$MCC=\frac{TP*TN-FP*FN}{\surd (TP+FP)*(TP+FN)*(TN+FP)*(TN+FN)}$$

S2b–S2e Fig show the ROC and precision-recall curves by applying PremPDI on the “Prempdi” training/test set using different thresholds. S2f Fig depicts the basic summary of performance metrics, including AUC for ROC and precision-recall curves and MCC. The results show that threshold of 1.5 kcal mol^-1^ has the highest AUC-ROC of 0.91 and MCC of 0.61 in distinguishing deleterious and neutral mutations (S2b and S2f Fig). Threshold of 1 kcal mol^-1^ has the highest AUC-PR of 0.83 and its AUC-ROC and MCC is 0.84 and 0.58 respectively (S2d and S2f Fig). S2c and S2e Fig show that threshold of 1 kcal mol^-1^ classification has the best performance in the deleterious mutation prediction with less than 10% false positive rate and more than 50% precision. Here, we choose ΔΔ*G*_*exp*_ = 1 kcal mol^-1^ as the threshold to define deleterious effect, and it is also in agreement with SAMPDI method for classifying large and small effects [22]. Fig 1d shows the ROC curves for PremPDI and PremPDI (CV3) to distinguish deleterious and neutral effects using threshold of 1 kcal mol^-1^. Therefore, PremPDI classifies a mutation as deleterious if its predicted ΔΔ*G* is higher or equal to 1.10 kcal mol^-1^ (S3 Fig). This threshold corresponds to 14% FPR and 77% TPR which minimizes the value of error $\text{ER}=\sqrt{{\left(1-TPR\right)}^{2}+FPR^{2}}$ to compensate retrieval sensitivity and specificity.

### Comparison of PremPDI with other methods

We compared our method with the other two available machine learning methods, mCSM-NA [21] and SAMPDI [22]. mCSM-NA uses graph-based signatures to calculate the changes in protein-nucleic acid binding affinity upon single mutations. SAMPDI uses a combination of modified MM/PBSA based energy terms with additional knowledge-based terms to predict the ΔΔ*G* values of interfacial mutations for protein-DNA complexes. The training sets for parameterizing PremPDI method and the other two have some differences, which is shown in S1 Table. Among 219 mutations from 49 complexes in PremPDI training set (“Prempdi”), 105 mutations from 16 complexes overlap with mCSM-NA training set of “Mcsm” (the overlapped set is named as “P.O.M”) and 77 mutations from 11 complexes overlap with SAMPDI training set of “Sampdi” (the overlapped set is named as “P.O.S”). 114 mutations from 33 complexes in “Prempdi” are not included in the “Mcsm” (named as “P.D.M”) and 142 mutations from 43 complexes in “Prempdi” are not in the “Sampdi” (named as “P.D.S”). Since SAMPDI is used in particular for interfacial mutations, we created a subset of “P.D.S” and named it as “P.D.S.I” that includes 77 interfacial mutations from 32 complexes.

**Fig 2:**
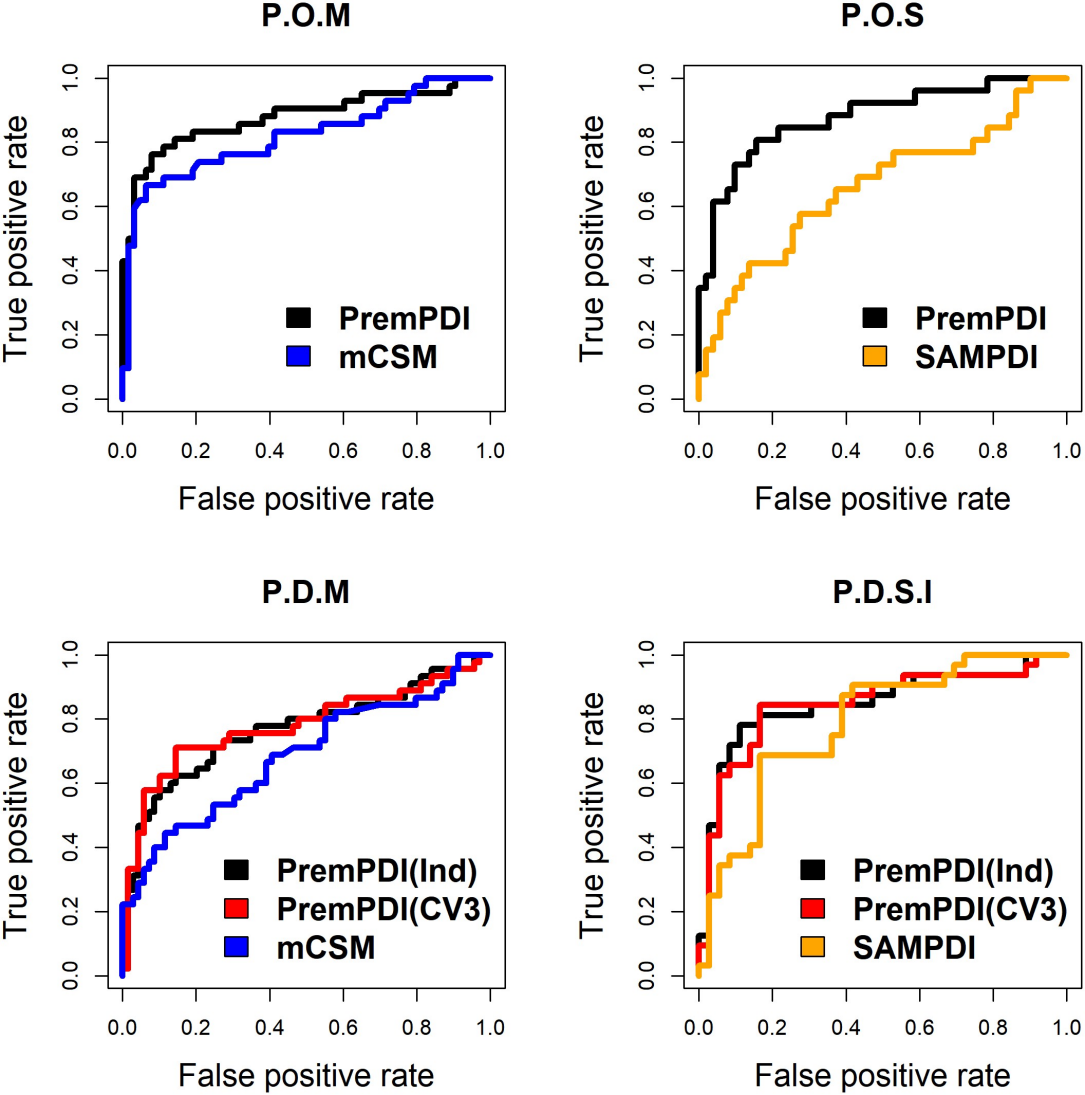
Assessment of classification performance between deleterious and neutral mutations. ROC curves for PremPDI, mCSM-NA and SAMPDI methods applied on different training and test set. More information is shown in Table 3.

We performed several types of comparisons between our method and the other two using four different test sets. “P.O.M” or “P.O.S” is the test set of overlapped mutations used for developing PremPDI and mCSM or SAMPDI respectively. So, we compared PremPDI with them using the model that built on the whole ‘Prempdi’ dataset. “P.D.M” or “P.D.S.I” test set represents the mutations that are included in the ‘Prempdi’ but not in the ‘Mcsm’ or ‘Sampdi’. So, to be fair, we used both “leave-one-complex-out” (CV3) results and the model built on the independent ‘P.O.M’ or ‘Prempdi-P.D.S.I’ dataset (named as PremPDI(Ind)) to compare with the other methods respectively. Pearson correlation coefficients and RMSE between experimental measurements (ΔΔ*G*_*exp*_) and predictions show that PremPDI presents a similar performance with mCSM-NA method and performs better than SAMPDI in predicting quantitative values of ΔΔG (Table 3). ROC curves shown in Fig 2 and AUC-ROC, AUC-PR and MCC values presented in Table 3 (The number of mutations in each category is shown in S4 Fig) demonstrate that the performance of PremPDI is notable in estimating deleterious effects (highly destabilizing) for all test sets and better than mCSM-NA and SAMPDI methods.

**Table 3:** Comparison of methods’ performances on different test sets.

Test set	Training set	Method	R	RMSE (kcal mol^-1^)	AUC-ROC	AUC-PR	MCC
P.O.M	Prempdi	PremPDI	0.80	0.81	0.88	0.87	0.54
	Mcsm	mCSM	0.76	0.95	0.82	0.79	0.50
P.O.S	Prempdi	PremPDI	0.68	0.63	0.88	0.81	0.52
	Sampdi	SAMPDI	0.39	0.80	0.66	0.53	0.27
P.D.M	Prempdi	PremPDI(CV3)	0.51	0.97	0.78	0.72	0.54
	P.O.M	PremPDI(Ind)	0.51	1	0.77	0.72	0.41
	Mcsm	mCSM	0.54	1.17	0.69	0.65	0.28
P.D.S.I	Prempdi	PremPDI(CV3)	0.70	1.10	0.85	0.82	0.62
	Prempdi- P.D.S.I	PremPDI(Ind)	0.74	1.08	0.85	0.83	0.68
	Sampdi	SAMPDI	0.53	1.32	0.79	0.71	0.35

R: Pearson correlation coefficient between experimental and predicted ΔΔ*G* values. RMSE: root-mean square error. AUC-ROC: the AUC values of ROC curves. AUC-PR: the AUC values of Precision-recall curves. MCC: Matthews correlation. All correlation coefficients are statistically significantly different from zero (p-value < 0.01). The descriptions of training and test set are shown in S1 Table. Nine mutations do not have SAMPDI scores in the P.D.S.I test set, so they were excluded in the comparison.

### Webserver input

The main requirement of the webserver is the 3D structure of a protein-DNA complex. The users can either input PDB code of the complex, then structures of either biological assemblies or asymmetric unit will be retrieved from the Protein Data Bank, or they can upload their own file with atomic coordinates. In either case, the structure file should contain at least two chains.

After the structure was retrieved correctly, the server will display a 3D view of the complex colored by chains or partners using the GLmol software. Each chain is listed with the corresponding protein or nucleic acid name. At the second step, two interacting partners should be defined. The user can assign one or multiple chains to either Partner 1 or Partner 2, but both partners should include at least one chain. Here, we restrict Partner 1 to proteins and Partner 2 to DNA and the selected protein/DNA chain will be put into the box of Partner1/Partner2 automatically. Only the selected chains of two partners will be taken into account during the calculation. If the interface size between two partners is more than 100 Å^2^, we define them interacting with each other and then perform the calculation. Interface size is calculated as the difference between the solvent accessible surface areas of complex and unbound partners.

**Fig 3:**
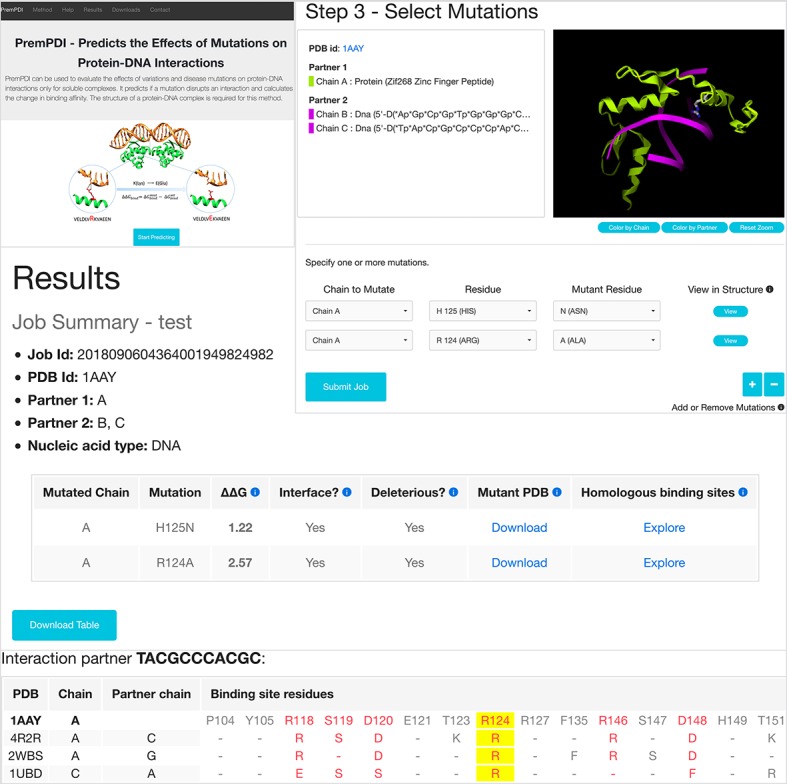
Left corner: The entry page of PremPDI server; right corner: The third step for selecting mutations, wild-type residue (R124) in the mutated site is shown in the 3D viewer; and bottom: Final results table and alignment of homologous binding sites.

The third step is to select mutations (Fig 3). Each mutation will be treated independently and up to 16 single mutations can be selected for one submission. After the chain and the mutated residue are selected, they can be visualized in the wild-type complex using the 3D viewer.

### Webserver output

For each mutation of a protein-DNA complex, PremPDI server provides the following results:

- ΔΔG (kcal mol^-1^), predicted binding affinity change induced by single mutation. Positive and negative signs correspond to destabilizing and stabilizing mutations predicted to decrease and increase binding affinity respectively.
- Interface (yes/no), PremPDI defines a residue to be located on the protein-DNA interface if residue’s solvent accessibility in the complex is lower than in the corresponding unbound partners.
- Deleterious (yes/no), PremPDI classifies a mutation as deleterious if ΔΔ*G* is higher or equal to 1.10 kcal mol^-1^. This threshold corresponds to a minimum value of ER to compensate retrieval sensitivity and specificity.
- Coordinates of the minimized mutant structure are provided for download.
- Protein binding sites in protein-DNA complexes homologous to the query are identified using Inferred Biomolecular Interactions Server at NCBI (IBIS) server [39]. It allows testing mutations of aligned binding site residues in homologous protein-DNA in PremPDI.

Results can be viewed directly on the browser (Fig 3) or downloaded as a plain text file.

## Supporting information

S1 Fig The number of mutations for each protein-DNA complex. (DOCX)

S2 Fig Assessment of classification performance between deleterious and neutral mutations by applying PremPDI on “Prempdi” dataset using different thresholds. (a) The definition and the number of deleterious, neutral and stabilizing mutations for four thresholds. (b) ROC curves. (c) shows the ROC curves corresponding to FPR less than 10%. (d) Precision-recall curves. (e) shows the precision-recall curves corresponding to precision over 50%. (f) The AUC values of ROC curves and Precision-recall curves, and Matthews correlation (MCC) for four thresholds. The best performance is shown in bold font.
(DOCX)

S3 FigROC curve for predicting deleterious mutations by applying PremPDI on the training set of “Prempdi”.Red point corresponds to the minimization of the value of error $\text{ER}=\sqrt{{\left(1-TPR\right)}^{2}+FPR^{2}}$.(DOCX)

S4 Fig The number of deleterious, neutral and stabilizing mutations for four datasets of P.O.M, P.O.S, P.D.M and P.D.S.I. Nine mutations do not have SAMPDI scores in the P.D.S.I test set, so they were excluded in the comparison.
(DOCX)

S1 Table The number of mutations in different data sets. (DOCX)

S2 Table Correlation between predicted and experimental values of ΔΔG for different structure optimization protocols. All calculations were performed by PremPDI energy function. “Prempdi-dbAMEPNI” includes 126 mutations and the mutations from dbAMEPNI database were not included in it. R: Pearson correlation coefficient between experimental and predicted ΔΔG values, and RMSE: root-mean squared error.
(DOCX)

S3 Table Features considered in model selection. (DOCX)

S4 Table PremPDI performance using different dielectric constants for protein interior in the PB calculation. (DOCX)

S5 Table PremPDI performance. (DOCX)

S6 Table Correlation matrixes and variance inflation factors (VIF) for the energy features in PremPDI. Correlation coefficients that are greater than 0.5 are underlined. Only correlation coefficients that are statistically significantly different from zero (P-value < 0.01) are shown.
(DOCX)

S7 Table Average weighting coefficients and corresponding standard deviation (in brackets) for all energy features in “CV1”, “CV2” and “CV3” cross-validation respectively. The weighting coefficients from the final PremPDI model were also shown for the comparison.
(DOCX)
