# Supplementary material for: PremPDI estimates and interprets the effects of missense mutations on protein-DNA interactions
Source: PLoS Comput Biol. 2018 Dec 11;14(12):e1006615. doi: 10.1371/journal.pcbi.1006615 (PMC6303081; doi:10.1371/journal.pcbi.1006615)
Supplement: S1 Fig — (DOCX) [file pcbi.1006615.s001.docx]

**Fig S1. The number of mutations for each protein-DNA complex.**
