# Supplementary material for: PremPDI estimates and interprets the effects of missense mutations on protein-DNA interactions
Source: PLoS Comput Biol. 2018 Dec 11;14(12):e1006615. doi: 10.1371/journal.pcbi.1006615 (PMC6303081; doi:10.1371/journal.pcbi.1006615)
Supplement: S2 Fig — (a) The definition and the number of deleterious, neutral and stabilizing mutations for four thresholds. (b) ROC curves. (c) shows the ROC curves corresponding to FPR less than 10%. (d) Precision-recall curves. (e) shows the precision-recall curves corresponding to precision over 50%. (f) The AUC values of ROC curves and Precision-recall curves, and Matthews correlation (MCC) for four thresholds. The best performance is shown in bold font. (DOCX) [file pcbi.1006615.s002.docx]

**a**

| Category | Definition | # of mutations |
| --- | --- | --- |
| **Deleterious** | $\Delta\Delta G_{exp}$(kcal mol^-1^) >= 1 | 87 |
|  | $\Delta\Delta G_{exp}$(kcal mol^-1^) >= 1.5 | 56 |
|  | $\Delta\Delta G_{exp}$(kcal mol^-1^) >= 2 | 46 |
|  | $\Delta\Delta G_{exp}$(kcal mol^-1^) >= 2.5 | 29 |
| **Neutral** | $\vert\Delta\Delta G_{exp}\vert$ (kcal mol^-1^) < 1 | 125 |
|  | $\vert\Delta\Delta G_{exp}\vert$ (kcal mol^-1^) < 1.5 | 156 |
|  | $\vert\Delta\Delta G_{exp}\vert$ (kcal mol^-1^) < 2 | 171 |
|  | $\vert\Delta\Delta G_{exp}\vert$ (kcal mol^-1^) < 2.5 | 190 |
| **Stabilizing** | $\Delta\Delta G_{exp}$(kcal mol^-1^) <= -1 | 7 |
|  | $\Delta\Delta G_{exp}$(kcal mol^-1^) <= -1.5 | 7 |
|  | $\Delta\Delta G_{exp}$(kcal mol^-1^) <= -2 | 2 |
|  | $\Delta\Delta G_{exp}$(kcal mol^-1^) <= -2.5 | 0 |


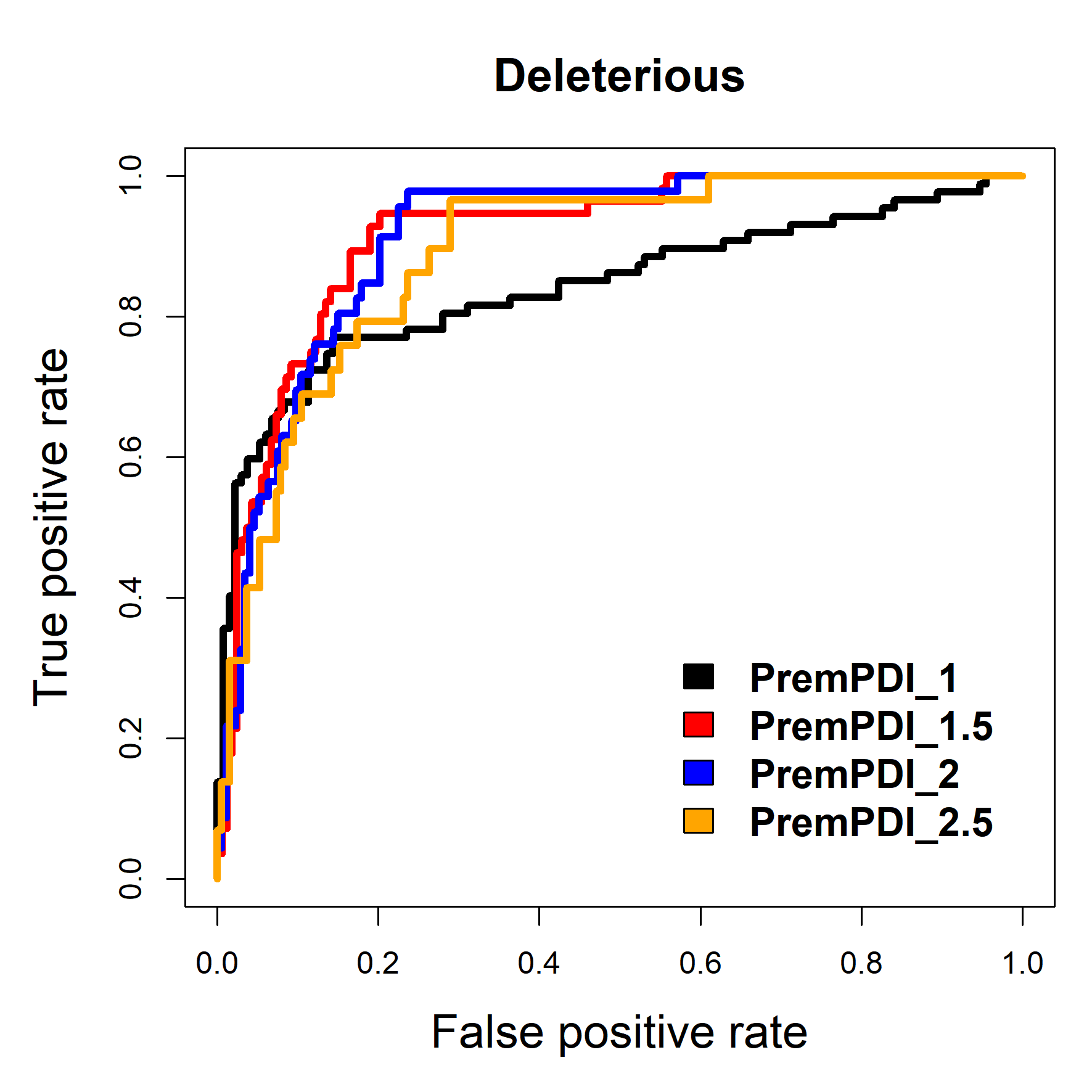
**b c**


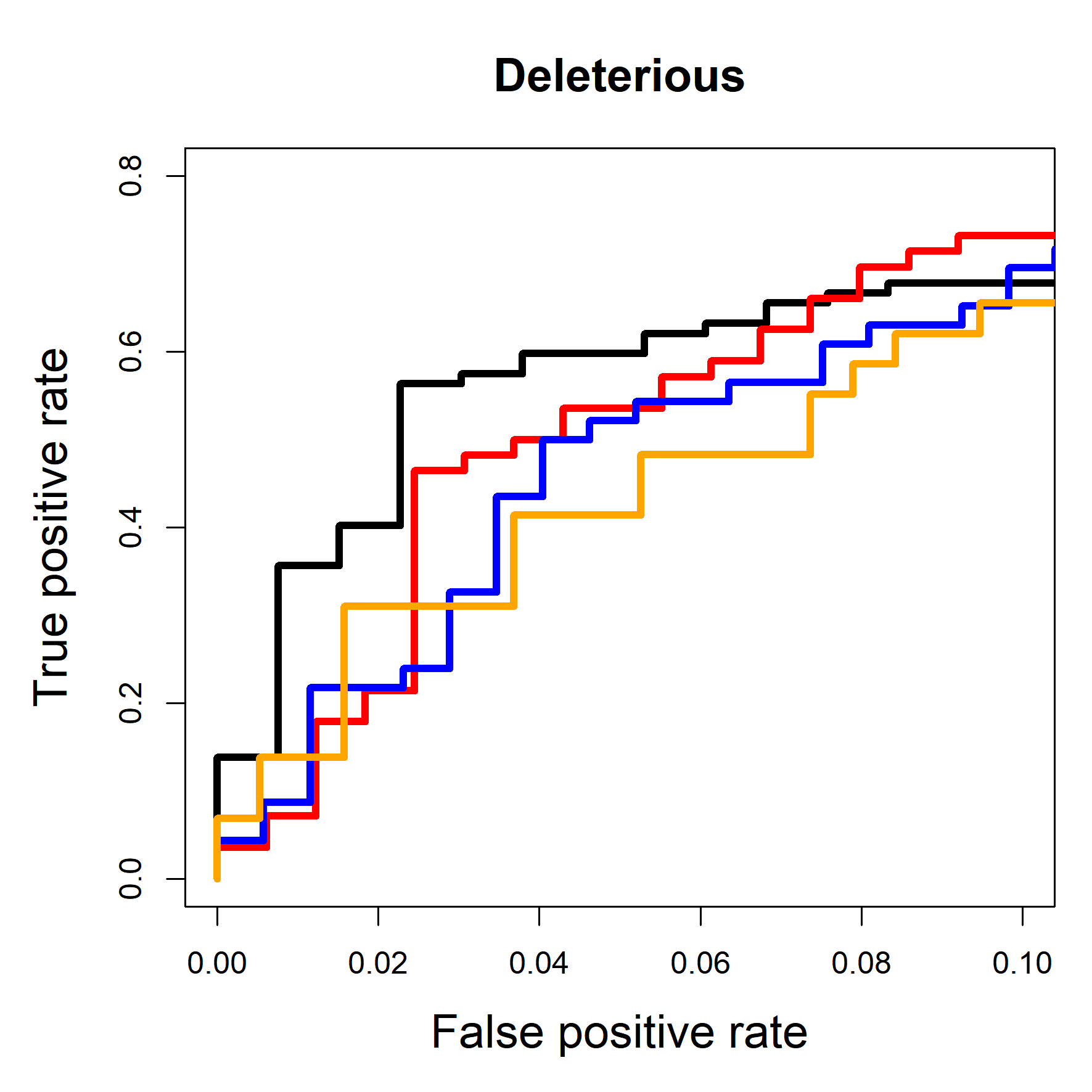


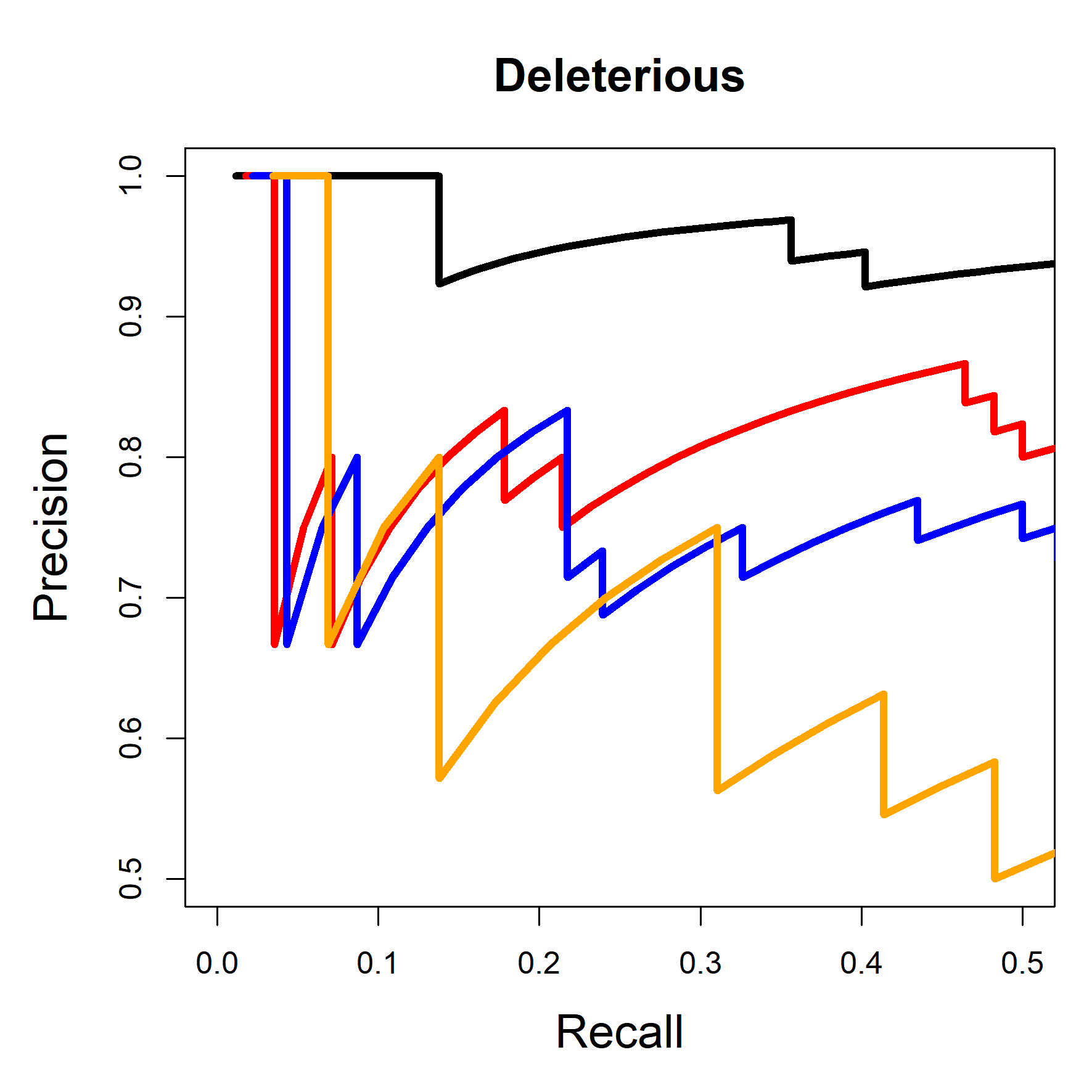
 **d e**


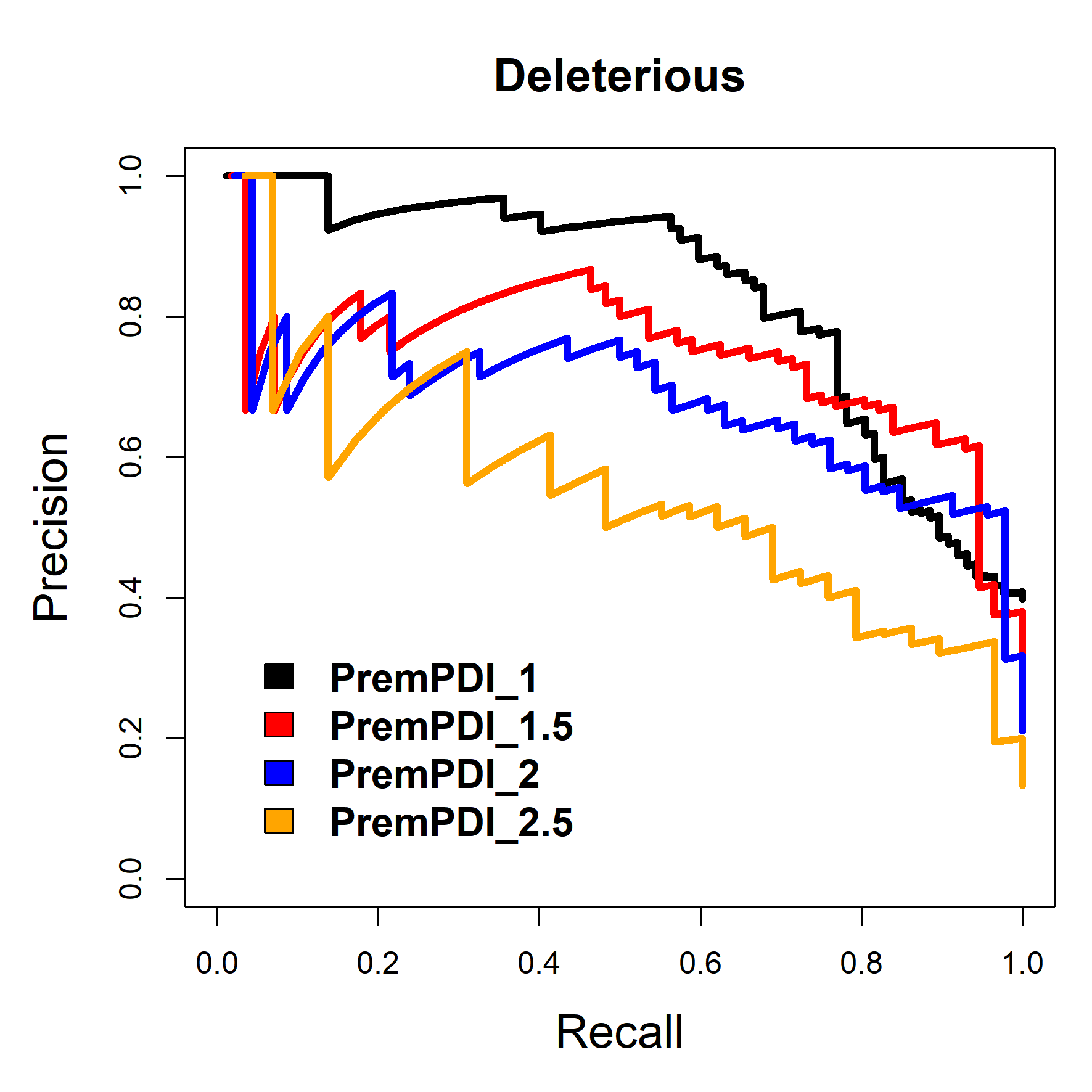


**f**

| Deleterious | AUC-ROC | AUC-PR | Matthews correlation |
| --- | --- | --- | --- |
| $\Delta\Delta G_{exp}$(kcal mol-1) >= 1 | 0.84 | **0.83** | 0.58 |
| $\Delta\Delta G_{exp}$(kcal mol-1) >= 1.5 | **0.91** | 0.74 | **0.61** |
| $\Delta\Delta G_{exp}$(kcal mol-1) >= 2 | 0.91 | 0.67 | 0.54 |
| $\Delta\Delta G_{exp}$(kcal mol-1) >= 2.5 | 0.89 | 0.54 | 0.44 |

**Fig S2. Assessment of classification performance between deleterious and neutral mutations by applying PremPDI on “Prempdi” dataset using different thresholds. (a) The definition and the number of deleterious, neutral and stabilizing mutations for four thresholds. (b) ROC curves. (c) shows the ROC curves corresponding to FPR less than 10%. (d) Precision-recall curves. (e) shows the precision-recall curves corresponding to precision over 50%. (f) The AUC values of ROC curves and Precision-recall curves, and Matthews correlation (MCC) for four thresholds. The best performance is shown in bold font.**
