# Supplementary material for: PremPDI estimates and interprets the effects of missense mutations on protein-DNA interactions
Source: PLoS Comput Biol. 2018 Dec 11;14(12):e1006615. doi: 10.1371/journal.pcbi.1006615 (PMC6303081; doi:10.1371/journal.pcbi.1006615)
Supplement: S3 Fig — Red point corresponds to the minimization of the value of error ER=(1−TPR)2+FPR2. (DOCX) [file pcbi.1006615.s003.docx]

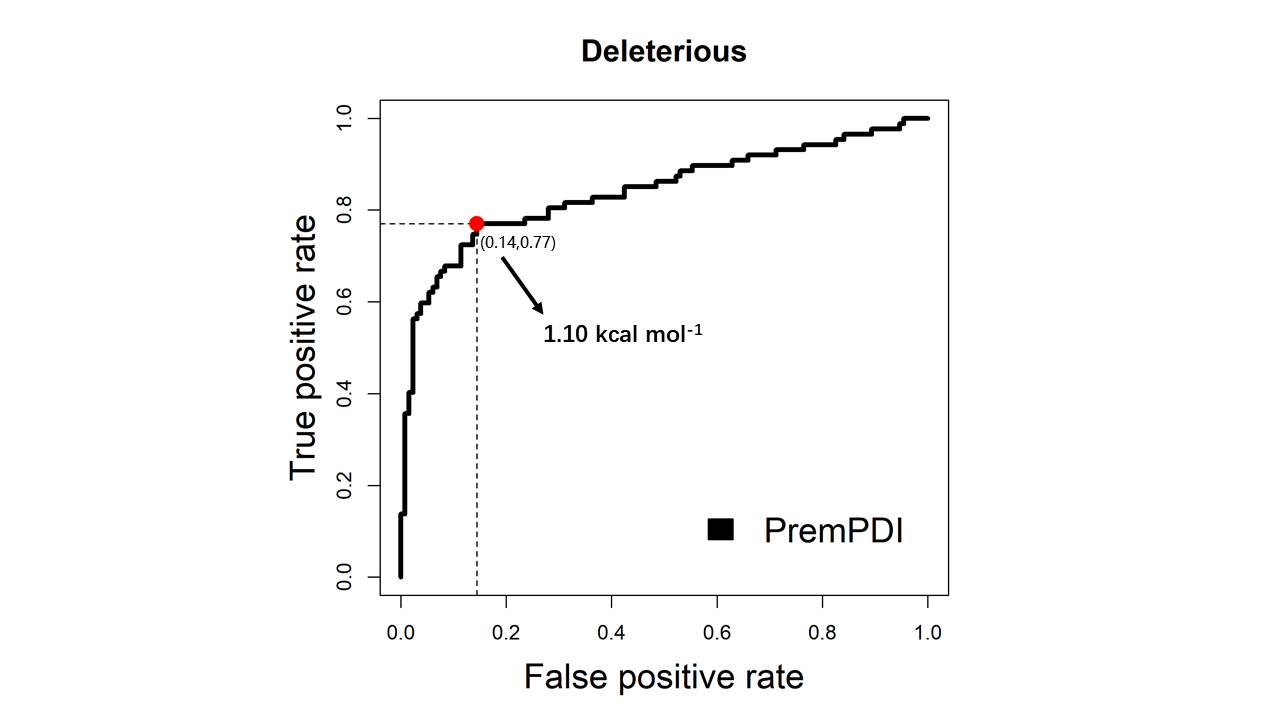


**Fig S3. ROC curve for predicting deleterious mutations by applying PremPDI on the training set of “Prempdi”. Red point corresponds to the minimization of the value of error ER=**$\sqrt{\boldsymbol{(1-TPR)}^{\boldsymbol{2}}\mathbf{+}\boldsymbol{FPR}^{\boldsymbol{2}}}$**.**
