# Supplementary material for: PremPDI estimates and interprets the effects of missense mutations on protein-DNA interactions
Source: PLoS Comput Biol. 2018 Dec 11;14(12):e1006615. doi: 10.1371/journal.pcbi.1006615 (PMC6303081; doi:10.1371/journal.pcbi.1006615)
Supplement: S4 Fig — Nine mutations do not have SAMPDI scores in the P.D.S.I test set, so they were excluded in the comparison. (DOCX) [file pcbi.1006615.s004.docx]

| Category | Definition | # of mutations | | | |
| --- | --- | --- | --- | --- | --- |
|  |  | P.O.M | P.O.S | P.D.M | P.D.S.I |
| **Deleterious** | $\Delta\Delta G_{exp}$(kcal mol^-1^) >= 1 | 42 | 26 | 45 | 32 |
| **Neutral** | $\vert\Delta\Delta G_{exp}\vert$ (kcal mol^-1^) < 1 | 56 | 51 | 69 | 32 |
| **Stabilizing** | $\Delta\Delta G_{exp}$(kcal mol^-1^) <= -1 | 7 | 0 | 0 | 4 |

**Fig S4. The number of deleterious, neutral and stabilizing mutations for four datasets of P.O.M, P.O.S, P.D.M and P.D.S.I. Nine mutations do not have SAMPDI scores in the P.D.S.I test set, so they were excluded in the comparison.**
