# Supplementary material for: PremPDI estimates and interprets the effects of missense mutations on protein-DNA interactions
Source: PLoS Comput Biol. 2018 Dec 11;14(12):e1006615. doi: 10.1371/journal.pcbi.1006615 (PMC6303081; doi:10.1371/journal.pcbi.1006615)
Supplement: S1 Table — (DOCX) [file pcbi.1006615.s005.docx]

**Table S1. The number of mutations in different data sets.**

| Dataset | # of complexes | # of mutations |
| --- | --- | --- |
| Prempdi | 49 | 219 |
| Mcsm | 38 | 331 |
| Mcsm.DNA | 33 | 264 |
| Sampdi | 13 | 105 |
| P.O.M | 16 | 105 |
| P.O.S | 11 | 77 |
| P.D.M | 33 | 114 |
| P.D.S | 43 | 142 |
| P.D.S.I | 32 | 77 |

‘Prempdi’ is the training set used for parameterizing PremPDI model. ‘Mcsm’ and ‘Sampdi’ are the training sets used for constructing mCSM-NA and SAMPDI model, respectively. ‘Mcsm.DNA’ is the set of mutations for protein-DNA complexes. ‘P.O.M’ or ‘P.O.S’ is the set of overlapped mutations between ‘Prempdi’ and ‘Mcsm’ or ‘Sampdi’. ‘P.D.M’ or ‘P.D.S’ indicates the mutations included in the ‘Prempdi’ but not in the ‘Mcsm’ or ‘Sampdi’ dataset. ‘P.D.S.I’ is the interfacial mutations.
