# Supplementary material for: PremPDI estimates and interprets the effects of missense mutations on protein-DNA interactions
Source: PLoS Comput Biol. 2018 Dec 11;14(12):e1006615. doi: 10.1371/journal.pcbi.1006615 (PMC6303081; doi:10.1371/journal.pcbi.1006615)
Supplement: S2 Table — All calculations were performed by PremPDI energy function. “Prempdi-dbAMEPNI” includes 126 mutations and the mutations from dbAMEPNI database were not included in it. R: Pearson correlation coefficient between experimental and predicted ΔΔG values, and RMSE: root-mean squared error. (DOCX) [file pcbi.1006615.s006.docx]

**Table S2. Correlation between predicted and experimental values of ΔΔG for different structure optimization protocols. All calculations were performed by PremPDI energy function.** “Prempdi-dbAMEPNI” includes 126 mutations and the mutations from dbAMEPNI database were not included in it. R: Pearson correlation coefficient between experimental and predicted ΔΔG values, and RMSE: root-mean squared error.

| Training/  Test set | FoldX  Repair | Minimization step |  | Restrained backbone | R | RMSE  (kcal mol^-1^) |
| --- | --- | --- | --- | --- | --- | --- |
| Prempdi- dbAMEPNI | Yes | 100 |  | Yes | 0.75 | 0.82 |
|  | **No** | **100** |  | **Yes** | **0.81** | **0.73** |
|  | No | 100 |  | No | 0.78 | 0.78 |
|  | No | 500 |  | Yes | 0.77 | 0.80 |
|  | No | 1000 |  | Yes | 0.76 | 0.81 |
|  | No | 1000 |  | No | 0.78 | 0.79 |
| Prempdi | Yes | 100 |  | Yes | 0.61 | 0.97 |
|  | **No** | **100** |  | **Yes** | **0.71** | **0.86** |

“FoldX Repair”: three-time structure relaxation on both wild-type and mutant Protein-DNA complex using RepairPDB module of FoldX. “Minimization step”: energy minimization in the gas phase was carried out for both wild type and mutant. “Restrained backbone”: harmonic restraints (with the force constant of 5 kcal mol-1 Å-2) applied on the backbone atoms of all residues. The best performance is shown in bold font.
