# Supplementary material for: PremPDI estimates and interprets the effects of missense mutations on protein-DNA interactions
Source: PLoS Comput Biol. 2018 Dec 11;14(12):e1006615. doi: 10.1371/journal.pcbi.1006615 (PMC6303081; doi:10.1371/journal.pcbi.1006615)
Supplement: S3 Table — (DOCX) [file pcbi.1006615.s007.docx]

**Table S3. Features considered in model selection**

| **Features** |
| --- |
| Amino acid substitution indexes and statistical protein contact potentials from AAindex database (<http://www.genome.jp/aaindex/>) |
| Van der Waals interaction energy |
| Electrostatic interaction energy |
| Polar solvation energy |
| Solvent accessible surface area |
| Energy terms from FoldX |
| Van der Waals repulsive energy |
| The length of protein, mutated chain and nucleic acid |
| Number of hydrogen bonds |
| Number of Salt-bridge |
| Classification of residue types |
| Configurational entropy of mutated residue |
| Hydrophobicity of mutated residue |
| Conservation score of the mutated site |
| Location of the mutated site |
| Secondary structure elements |
