# Supplementary material for: PremPDI estimates and interprets the effects of missense mutations on protein-DNA interactions
Source: PLoS Comput Biol. 2018 Dec 11;14(12):e1006615. doi: 10.1371/journal.pcbi.1006615 (PMC6303081; doi:10.1371/journal.pcbi.1006615)
Supplement: S4 Table — (DOCX) [file pcbi.1006615.s008.docx]

**Table S4. PremPDI performance using different dielectric constants for protein interior in the PB calculation.**

|  | Dielectric constants | R | RMSE  (kcal mol^-1^) |
| --- | --- | --- | --- |
|  | **2** | **0.71** | **0.86** |
|  | 6 | 0.70 | 0.87 |
|  | 10 | 0.69 | 0.88 |
|  | 14 | 0.69 | 0.88 |
|  | 18 | 0.69 | 0.89 |
|  | 20 | 0.69 | 0.89 |

The best performance is shown in bold font.
