# Supplementary material for: PremPDI estimates and interprets the effects of missense mutations on protein-DNA interactions
Source: PLoS Comput Biol. 2018 Dec 11;14(12):e1006615. doi: 10.1371/journal.pcbi.1006615 (PMC6303081; doi:10.1371/journal.pcbi.1006615)
Supplement: S5 Table — (DOCX) [file pcbi.1006615.s009.docx]

**Table S5. PremPDI performance.**

| Test set  (# of mutations) | Method | R | RMSE  (kcal mol^-1^) | Slope |
| --- | --- | --- | --- | --- |
| Prempdi  (219) | PremPDI | 0.71 | 0.86 | 1 |
|  | PremPDI (CV1) | 0.68 | 0.90 | 0.94 |
|  | PremPDI (CV2) | 0.68 | 0.90 | 0.95 |
|  | PremPDI (CV3) | 0.63 | 0.95 | 0.90 |
| Alanine-scanning mutations  (179) | PremPDI | 0.68 | 0.87 | 0.97 |
|  | PremPDI (CV1) | 0.64 | 0.91 | 0.90 |
|  | PremPDI (CV2) | 0.64 | 0.91 | 0.90 |
|  | PremPDI (CV3) | 0.58 | 0.96 | 0.87 |
| Non-Alanine- scanning mutations  (40) | PremPDI | 0.64 | 0.81 | 0.88 |
|  | PremPDI (CV1) | 0.60 | 0.85 | 0.82 |
|  | PremPDI (CV2) | 0.61 | 0.85 | 0.83 |
|  | PremPDI (CV3) | 0.58 | 0.88 | 0.72 |
| Interfacial mutations (134) | PremPDI | 0.71 | 0.86 | 1.01 |
|  | PremPDI (CV1) | 0.68 | 0.90 | 0.96 |
|  | PremPDI (CV2) | 0.68 | 0.90 | 0.96 |
|  | PremPDI (CV3) | 0.64 | 0.95 | 0.89 |
| Non-interfacial mutations  (85) | PremPDI | 0.69 | 0.85 | 0.98 |
|  | PremPDI (CV1) | 0.64 | 0.91 | 0.91 |
|  | PremPDI (CV2) | 0.64 | 0.91 | 0.91 |
|  | PremPDI (CV3) | 0.59 | 0.95 | 0.91 |

R: Pearson correlation coefficient between experimental and predicted ΔΔG values. RMSE: root-mean square error. The last column shows the slope of the regression line between experimental and predicted ΔΔG values. All correlation coefficients are statistically significantly different from zero (P-value << 0.01).
