# Supplementary material for: PremPDI estimates and interprets the effects of missense mutations on protein-DNA interactions
Source: PLoS Comput Biol. 2018 Dec 11;14(12):e1006615. doi: 10.1371/journal.pcbi.1006615 (PMC6303081; doi:10.1371/journal.pcbi.1006615)
Supplement: S6 Table — Correlation coefficients that are greater than 0.5 are underlined. Only correlation coefficients that are statistically significantly different from zero (P-value < 0.01) are shown. (DOCX) [file pcbi.1006615.s010.docx]

**Table S6. Correlation matrixes and variance inflation factors (VIF) for the energy features in PremPDI. Correlation coefficients that are greater than 0.5 are underlined.** Only correlation coefficients that are statistically significantly different from zero (P-value < 0.01) are shown.

| **Pearson Correlation matrixes** | | | | | | | | |
| --- | --- | --- | --- | --- | --- | --- | --- | --- |
|  | $\Delta\Delta G_{solv}$ | $\Delta E_{fold}$ | ${\Delta N}_{Hbond}^{p1-p2}$ | $\Delta_{location}^{mut}$ | ${SA}_{{com}/p2}^{wt}$ | $N_{Hbond}^{wt.\left( site-all \right)}$ | $\Delta E_{elec}^{mut.\left( p1-p2 \right)}$ | $L_{mut}$ |
| $\Delta E_{fold}$ | 0.27 |  |  |  |  |  |  |  |
| ${\Delta N}_{Hbond}^{p1-p2}$ | -0.71 |  |  |  |  |  |  |  |
| $\Delta_{location}^{mut}$ | 0.20 |  | -0.23 |  |  |  |  |  |
| ${SA}_{{com}/p2}^{wt}$ |  |  | 0.21 | -0.38 |  |  |  |  |
| $N_{Hbond}^{wt.\left( site-all \right)}$ | 0.27 | 0.35 | -0.28 |  |  |  |  |  |
| $\Delta E_{elec}^{mut.\left( p1-p2 \right)}$ |  |  |  |  |  |  |  |  |
| $L_{mut}$ | -0.25 |  | 0.26 | -0.30 | 0.74 |  |  |  |
| $\Delta\Delta E_{vdw}^{site-all}$ |  |  |  |  |  |  |  |  |

| **Variance inflation factors (VIF)** | | | | | | | | |
| --- | --- | --- | --- | --- | --- | --- | --- | --- |
| $\Delta\Delta G_{solv}$ | $\Delta E_{fold}$ | ${\Delta N}_{Hbond}^{p1-p2}$ | $\Delta_{location}^{mut}$ | ${SA}_{{com}/p2}^{wt}$ | $N_{Hbond}^{wt.\left( site-all \right)}$ | $\Delta E_{elec}^{mut.\left( p1-p2 \right)}$ | $L_{mut}$ | $\Delta\Delta E_{vdw}^{site-all}$ |
| 2.26 | 1.34 | 2.16 | 1.23 | 2.51 | 1.29 | 1.15 | 2.50 | 1.11 |
