# Supplementary material for: PremPDI estimates and interprets the effects of missense mutations on protein-DNA interactions
Source: PLoS Comput Biol. 2018 Dec 11;14(12):e1006615. doi: 10.1371/journal.pcbi.1006615 (PMC6303081; doi:10.1371/journal.pcbi.1006615)
Supplement: S7 Table — The weighting coefficients from the final PremPDI model were also shown for the comparison. (DOCX) [file pcbi.1006615.s011.docx]

**Table S7. Average weighting coefficients and corresponding standard deviation (in brackets) for all energy features in “CV1”, “CV2” and “CV3” cross-validation respectively. The weighting coefficients from the final PremPDI model were also shown for the comparison.**

|  | CV1 | CV2 | CV3 | PremPDI |
| --- | --- | --- | --- | --- |
| Intercept | -1.01(0.21) | -1(0.11) | -1.01(0.04) | -1.01 |
| $\boldsymbol{SA}_{\boldsymbol{com/p}\boldsymbol{2}}^{\boldsymbol{wt}}$ | 0.24(0.04) | 0.24(0.02) | 0.24(9.4e-3) | 0.24 |
| $\boldsymbol{\Delta\Delta}\boldsymbol{G}_{\boldsymbol{solv}}$ | 3.84e-2(8.78e-3) | 3.73e-2(3.86e-3) | 3.74e-2(1.55e-3) | 3.75e-2 |
| $\boldsymbol{\Delta N}_{\boldsymbol{Hbond}}^{\boldsymbol{p}\boldsymbol{1-p}\boldsymbol{2}}$ | 0.2(0.04) | 0.2(0.02) | 0.2(0.01) | 0.2 |
| $\boldsymbol{\Delta}\boldsymbol{E}_{\boldsymbol{elec}}^{\boldsymbol{mut.}\left( \boldsymbol{p}\boldsymbol{1-p}\boldsymbol{2} \right)}$ | 9.89e-5(1.93e-5) | 9.68e-5(1.01e-5) | 9.57e-5(4.63e-6) | 9.55e-05 |
| $\boldsymbol{N}_{\boldsymbol{Hbond}}^{\boldsymbol{wt.(site-all)}}$ | 0.23(0.05) | 0.23(0.02) | 0.23(7.5e-3) | 0.24 |
| $\boldsymbol{L}_{\boldsymbol{mut}}$ | -2.75e-3(7.32e-4) | -2.75e-3(3.94e-4) | -2.82e-3(1.7e-4) | -2.82e-3 |
| $\boldsymbol{\Delta\Delta}\boldsymbol{E}_{\boldsymbol{vdw}}^{\boldsymbol{site-all}}$ | 0.07(0.02) | 0.06(0.01) | 0.06(3.22e-3) | 0.06 |
| $\boldsymbol{\Delta}\boldsymbol{E}_{\boldsymbol{fold}}$ | 0.86(0.35) | 0.9(0.15) | 0.89(0.06) | 0.89 |
| $\boldsymbol{\Delta}_{\boldsymbol{location}}^{\boldsymbol{mut}}$ | 0.39(0.16) | 0.4(0.06) | 0.42(0.03) | 0.42 |
